# New nematicidal and antimicrobial secondary metabolites from a new species in the new genus, *Pseudobambusicola thailandica*

**DOI:** 10.3897/mycokeys.33.23341

**Published:** 2018-03-22

**Authors:** Zeljka Rupcic, Clara Chepkirui, Margarita Hernández-Restrepo, Pedro W. Crous, Janet Jennifer Luangsa-ard, Marc Stadler

**Affiliations:** 1 Department Microbial Drugs, Helmholtz Centre for Infection Research GmbH, Inhoffenstraße 7, 38124 Braunschweig, Germany; 2 German Centre for Infection Research (DZIF), partner site Hannover-Braunschweig, 38124 Braunschweig, Germany; 3 Westerdijk Fungal Biodiversity Institute, P.O. Box 85167, 3508 AD Utrecht, The Netherlands; 4 National Centre for Genetic Engineering and Biotechnology (BIOTEC), Pathum Thani 12120, Thailand

**Keywords:** Antifungal agent, deoxyphomalone, monocerin, nematode-antagonism, nematicide, phylogeny

## Abstract

During the course of a study on the functional biodiversity of the mycobiota inhabiting rainforests in Thailand, a fungal strain was isolated from a plant sample and shown to represent an undescribed species, as inferred from a combination of morphological and molecular phylogenetic methods. Molecular phylogenetic analyses, based on four DNA loci, revealed a phylogenetic tree with the newly generated sequences clustering in a separate branch, together with members of the Sulcatisporaceae (Pleosporales, Ascomycota). The Thai specimen morphologically resembled *Neobambusicola strelitziae* in having pycnidial conidiomata with phialidic conidiogenous cells that produce both fusoid-ellipsoid macroconidia and subcylindrical microconidia. However, the new fungus, for which the name *Pseudobambusicola thailandica* is proposed, differs from *N. strelitziae* in having conidiomata with well-defined necks, the presence of globose to subglobose thick-walled cells adjacent to conidiomata and the production of chlamydospores in culture. When cultures of *P. thailandica*, growing on water agar, were confronted with *Caenorhabditis elegans* nematodes, worms approaching the fungal mycelia were killed. This observation gave rise to a study of its secondary metabolites and six novel and two known compounds were isolated from submerged cultures of *P. thailandica*. The structures of metabolites 1–6, for which the trivial names thailanones A–F are proposed, were elucidated using a combination of spectral methods, including extensive 1 and 2D NMR analysis and high resolution mass spectrometry. Compounds 4 and 8 showed strong nematicidal and weak antifungal activity, whereas all other tested compounds showed moderate to weak nematicidal activity but no significant effects in the serial dilution assay against various fungi and bacteria. Compounds 1 and 8 also inhibited growth of the pathogenic basidiomycete *Phellinus tremulae* in a plate diffusion assay.

## Introduction

Fungi are regarded as prolific sources of secondary metabolites with prominent and selective biological activities that can serve as a basis for development of new antimicrobials, agrochemical pesticides and other useful compounds (Bills and Gloer 2016, Karwehl and Stadler 2017). In particular, the mycobiota of tropical countries are widely unexplored and can still yield a plethora of novel chemical entities. In recent years, many novel compounds with, for example, antimicrobial (Helaly et al. 2016, 2017, Richter et al. 2016, Kuephadungphan et al. 2017), cytotoxic (Surup et al. 2017) and antioxidative (Kuhnert et al. 2015) effects were isolated in the authors’ laboratory from tropical fungi. Furthermore, fungi represent a rich source of nematicidal compounds because they are both prey and natural antagonists of nematodes. Thus, understanding the chemical basis for fungi nematode interactions offers natural biocontrol strategies (Anke et al. 1995). According to Degenkolb and Vilcinskas (2016), approximately 700 species of nematophagous fungi have been described so far and four ecophysiological categories have been proposed. However, little has been done to screen for metabolites in nematophagous fungi or, for that measure, nematicidal metabolites in other fungi since the first studies of this kind during the 1990s (Stadler et al. 1993a, b, 1994).

Environmentally compatible and low-cost alternatives to chemical control measures for phytoparasitic nematodes are urgently needed and these must not affect vertebrates, crops and other non-target organisms. Highly specific, preferably soil-borne antagonists are best suited for this purpose (Degenkolb and Vilcinskas 2016).

In this context, fungi isolated from nature were examined for morphological features and by ITS sequencing. The strains that turned out to belong to well-studied, ubiquitous mycotoxin-producing genera (in particular Trichocomaceae and Hypocreaeae) were discarded. Those strains that belong to less studied phylogenetic lineages were selected for studies of their antagonistic activities. They were first tested using a water agar assay to detect nematicidal effects and, in parallel, extracts were prepared and checked in an agar plate diffusion assay for antifungal and nematicidal activities. Herein, the authors report the discovery of a new genus and species *Pseudobambusicola thailandica* and its six novel and two known secondary metabolites, including their isolation, structure elucidation and biological activity.

## Materials and methods

### Fungal isolation

During a fungal exploration in Thailand in 2015, an unrecognised fungus was found growing on a twig of an unidentified plant. The twig was incubated in a damp chamber and treated according to Castañeda-Ruiz et al. (2016). Single conidial isolates were established from sporulating conidiomata in Petri dishes containing water agar (WA; Difco agar 5 g, tap water 1 l). Colonies were sub-cultured on potato carrot agar (PCA; potatoes 20 g; carrots 20 g; agar 20 g; distilled water 1 l) and oatmeal agar (OA; oatmeal 30 g; agar 18 g; distilled water 1 l) as described previously (Hernández-Restrepo et al. 2017). Herbarium type material and the ex-type strain are maintained at the BIOTEC Bangkok herbarium (BBH) and at the BIOTEC culture collection (BCC; both Pathum Thani, Thailand), respectively.

### Morphology

Morphological features were characterised from colonies growing on OA or on synthetic nutrient-poor agar (SNA; Nirenberg 1976) supplemented by fragments of autoclaved pine needles and incubated at 25 °C under continuous near-ultraviolet light to promote sporulation. Colony colours were assessed according to the charts of Rayner (1970). Micromorphological descriptions and measurements for 30 replicates of relevant features were carried out from mature conidiomata and conidia mounted in lactic acid 90%. Photomicrographs were made following Hernández-Restrepo et al. (2017).

### DNA isolation, amplification and sequences analyses

Genomic DNA was extracted from fungal colonies growing on MEA using the Wizard® Genomic DNA purification kit (Promega, Madison, USA) following the manufacturer’s protocols. The nuclear rDNA operon spanning the 3’ end of the 18S nrRNA gene, the first internal transcribed spacer (ITS1), the 5.8S nrRNA gene, the second ITS region (ITS2) and approximately 900 bp of the 5’ end of the large subunit of the nrRNA gene (LSU), part of the RNA polymerase II second largest subunit gene (*rpb2*) and part of the translation elongation factor 1-α gene (*tef1*) were amplified following Hernández-Restrepo et al. (2016). The programme SeqMan Pro v. 10.0.1 (DNASTAR, Madison, WI, USA) was used to obtain consensus sequences for each DNA region. Blast searches using ITS and LSU sequences were performed and the closest matches and related taxa were retrieved from GenBank and included in the phylogenetic analyses (Table 1, See Suppl. material 1). Alignments were produced with MAFFT v. 7 (Katoh and Standley 2013), checked and refined using MEGA v. 6 (Tamura et al. 2013) and SequenceMatrix (Vaidya et al. 2011). Individual alignments for each locus and the concatenated four-loci dataset were analysed by maximum likelihood (ML) with gamma model of rate heterogeneity using the RAxML HPC BlackBox v. 8.2.8 (Stamatakis 2014) online server of the Cipres Science gateway portal (Miller et al. 2010). The maximum likelihood search option was used to search for the best-scoring tree after bootstrapping. By default, the RAxML BlackBox calculates statistical support for branches by rapid bootstrap analyses of 1000 replicates (Stamatakis 2014). Bootstrap support (bs) values ≥ 70 % were considered significant. Incongruence amongst datasets was tested by visual inspection of all groups with ≥ 70 % bs in partial trees of each locus to search for potentially conflicting groups. A Markov Chain Monte Carlo (MCMC) algorithm was used to generate phylogenetic trees with Bayesian probabilities from the concatenated four-loci dataset using MrBayes v. 3.2.6 (Ronquist et al. 2012). Two analyses of four MCMC chains were run from random trees, trees were sampled every 100 generations and 25 % of them were discarded as the burn-in phase. Posterior probabilities (pp) were determined from the remaining trees. The sequences generated during this study and the alignments used in the phylogenetic analyses were deposited in GenBank and TreeBASE, respectively.

**Table 1.** Isolates and GenBank accession numbers used in the phylogenetic analyses.

Taxa	Strain number^1^	GenBank accession numbers^2^				References
		ITS	LSU	*rpb2*	*tef1*	
*Alternaria tenuissima*	CBS 918.96	–	KC584311	KC584435	KC584693	Woudenberg et al. 2013
*Bambusicola didymospora*	MFLUCC 10-0557	KU940116	KU863105	KU940163	KU940188	Dai et al. 2017
*B. loculata*	MFLU 15-0056	KP761732	KP761729	KP761715	KP761724	Dai et al. 2015
*B. pustulata*	MFLUCC 15-0190	KU940118	KU863107	KU940165	KU940190	Dai et al. 2017
*B. splendida*	MFLUCC 11-0611	KU940121	KU863110	KU940168	–	Dai et al. 2017
*Coniothyrium palmicola*	CBS 161.37	JX681086	JX681086	–	–	Verkley et al. 2014
*Dendrothyrium longisporum*	CBS 824.84	JX496115	JX496228	–	–	Verkley et al. 2014
*Dydimella exigua*	CBS 183.55	NR135936	EU754155	GU357800	KR184187	De Gruyter et al. 2009, Schoch et al. 2009, Kim et al. 2016
*Keissleriella culmifida*	KT 2308	–	AB807591	–	AB808570	Tanaka et al. 2015
*K. quadriseptata*	KT 2292	NR145135	AB807593	–	AB808572	Tanaka et al. 2015
*Latorua caligans*	CBS 576.65	NR132923	KR873266	–	–	Crous et al. 2015a
*Leptosphaeria doliolum*	CBS 505.75	JF740205	GQ387576	KY064035	GU349069	De Gruyter et al. 2013, Schoch et al. 2009
*Lophiostoma arundinis*	AFTOL-ID 1606	–	DQ782384	DQ782386	DQ782387	Schoch et al. 2009
*Macrodiplodiopsis desmazieri*	CBS 140062	NR132924	KR873272	–	–	Crous et al. 2015a
*Magnicamarosporium iriomotense*	KT 2822	AB809640	AB807509	–	AB808485	Tanaka et al. 2015
*Massarina phragmiticola*	CBS 110446	–	DQ813510	–	–	Kodsueb et al. 2007
*Montagnula bellevaliae*	MFLUCC 14-0924	KT443906	KT443902	–	–	Hongsanan et al. 2015
*M. scabiosae*	MFLUCC 14-0954	KT443907	KT443903	–	–	Hongsanan et al. 2015
*Murilentithecium clematidis*	MFLUCC 14-0562	KM408757	KM408759	KM454447	KM454445	Wanasinghe et al. 2014
*Neobambusicola strelitziae*	CBS 138869	NR 137945	KP004495	–	**MG976037**	Crous et al. 2014, this study
*Palmiascoma gregariascomum*	MFLUCC 11-0175	KP744452	KP744495	KP998466	–	Liu et al. 2015
*Parabambusicola bambusina*	H 4321	–	AB807536	–	AB808511	Tanaka et al. 2015
*Paraconiothyrium brasiliense*	CBS 122851	JX496036	JX496149	–	–	Verkley et al. 2014
*Phoma herbarum*	CBS 615.75	NR135967	EU754186	KP330420	KR184186	Aveskamp et al. 2009, Chen et al. 2015
*Pleurophoma ossicola*	CPC 24985	KR476737	KR476770	–	–	Crous et al. 2015b
*Polyschema congolensis*	CBS 542.73	–	EF204502	EF204486	–	Shenoy et al. 2010
*P. terricola*	CBS 301.65	–	EF204504	EF204487	–	Shenoy et al. 2010
*Pseudobambusicola thailandica* sp. nov.	BCC 79462	**MG926559**	**MG926560**	**MG926561**	**MG926562**	This study
*Pseudoleptosphaeria etheridgei*	CBS 125980	NR111620	JF740291	–	–	De Gruyter et al. 2013
*Pseudoxylomyces elegans*	KT 2887	–	AB807598	–	AB808576	Tanaka et al. 2015
*Setoseptoria arundinacea*	KT 552	–	AB807574	–	AB808550	Tanaka et al. 2015
*Stemphylium vesicarium*	CBS 191.86	KC584239	JX681120	KC584471	KC584731	Woudenberg et al. 2013
*Sulcatispora acerina*	KT 2982	LC014597	LC014610	–	LC014615	Tanaka et al. 2015
*S. berchemiae*	KT 1607	AB809635	AB807534	–	AB808509	Tanaka et al. 2015
*Trematosphaeria pertusa*	CBS 122368	NR132040	FJ201990	FJ795476	KF015701	Zhang et al. 2009

^1^ BCC: BIOTEC Culture Collection, Thailand; CBS: Westerdijk Fungal Biodiversity Institute, Utrecht, The Netherlands; CPC: Culture collection of Pedro Crous, housed at CBS; KT and H: Culture collection of K. Tanaka and K. Hirayama, housed at the National Institute of Agrobiological Science, Japan (MAFF); MFLU: Mae Fah Laung University Herbarium, Chiang Rai, Thailand; MFLUCC: Mae Fah Luang University Culture Collection, Chiang Rai, Thailand.

^2^ ITS: internal transcribed spacer regions 1 & 2 including 5.8S nrRNA gene; LSU: large subunit of the nrRNA gene, *rpb2*: partial RNA polymerase II second largest subunit gene; *tef1*: partial translation elongation factor 1-α gene. Sequences generated in the present study are in **bold.**

### Chromatography and spectral methods

1D and 2D nuclear magnetic resonance (NMR) spectra were recorded on a Bruker Avance III 700 spectrometer with a 5 mm TXI cryoprobe (^1^H 700 MHz, ^13^C 175 MHz) and a Bruker Avance III 500 (^1^H 500 MHz, ^13^C 125 MHz) spectrometer, UV spectra were recorded with a Shimadzu UV-2450 UV−Vis spectrophotometer and optical rotations were measured on a Perkin-Elmer 241 polarimeter. Analytical HPLC was carried out on an Agilent 1200 Series, equipped with degasser, binary pump SL, autosampler and connected to a diode array detection/light scattering detector Corona Ultra RS. A Waters C18 Acquity UPLC BEH column (2.1 × 50 mm, 1.7 μm) was used as stationary phase. The mobile phase consisted of H_2_O + 0.1% formic acid (solvent A) and acetonitrile + 0.1% formic acid (solvent B) with the following gradient: 0–0.5 min 5% B, 0.5–20 min 100% B, 20–30 min 100% B; injection volume was 2 µl, flow rate 600 µl/min.

HPLC-ESI-MS spectra were recorded on an ion trap mass spectrometer [scan range 100–2000 m/z, capillary voltage 4000 V, dry temperature 250 °C] (amaZon speed, Bruker) and HR-ESIMS spectra on a time-of-flight (TOF) MS [scan range 250–25000 m/z, capillary voltage 4500 V, dry temperature 200 °C] (MaXis, Bruker). In parallel, UV/Vis spectra in the range of 200–600 nm were recorded.

Chemicals and solvents were obtained from AppliChem GmbH (Darmstadt, Germany), Avantor Performance Materials (Deventer, Netherlands), Carl Roth GmbH & Co. KG (Karlsruhe, Germany) and Merck KGaA (Darmstadt, Germany) in analytical and HPLC grade.

### Fermentation and extraction

A seed culture was prepared as follows: five mycelial plugs (0.5 × 0.5 cm^2^) were cut from actively growing colonies maintained on YM 6.3 agar (malt extract 10 g/l, D-glucose 4 g/l, yeast extract 4 g/l, agar 20 g/l, pH 6.3 before autoclaving) and placed into a 500 mL Erlenmeyer flask containing 200 mL Q6½ medium (D-glucose 2.5 g/l, glycerol 10 g/l, cotton seed flour 5 g/l, pH 6.3) and incubated on a rotary shaker for 96 hours at 24 °C and 140 rpm. 20 mL of the seed culture were added into 10 × 1000 ml sterile Erlenmeyer flasks with 500 ml of Q6 ½ medium (5 l total) and incubated on a rotary shaker (288 hours, 24 °C, 140 rpm).

Biomass and supernatant were separated by means of centrifugation and filtration. The mycelia were extracted twice with acetone (2 l), the extract was evaporated *in vacuo* and the remaining aqueous phase extracted with equal amounts of ethyl acetate three times. One percent (1 %) of Amberlite XAD-16N was given to the culture broth and stirred for 1 h. After filtration, the XAD resin was extracted as described above. 220 mg and 88 mg of mycelial and supernatant crude extracts were obtained, respectively.

### Isolation of the compounds 1–8

The supernatant crude extract was fractionated on preparative HPLC (Gilson GX270 Series HPLC system). The reversed phase C18 column (Nucleodur 150/40, 10 µm, 110 Å; with a precolumn VP 100/10; Macherey-Nagel) was used as a stationary phase and the mobile phase was composed of deionised water + TFA 0.05 % (Milli-Q, Millipore, Schwalbach, Germany; solvent A) and acetonitrile (ACN) + TFA 0.05 % (solvent B). The fractionation was accomplished with the following gradient: 15 % of B isocratic for 5 min, followed by a linear increase to 80 % B over 30 min, afterwards increasing to 100% B in 5 min and thereafter isocratic conditions at 100 % for 5 min. In total, 7 compounds were obtained from the supernatant crude extract: Compound **1** (thailanone A; 1 mg) was obtained at the retention time t_R_ = 6 min, compound **2** (thailanone B; 1 mg) at t_R_ = 4.3 min, compound **3** (thailanone C; 1.3 mg) at t_R_ = 6.4 min, compound **4** (thailanone D; 1 mg) at t_R_ = 8.1 min; compound **5** (thailanone E; 4.2 mg) at t_R_ = 8.2, compound **6** (thailanone F; 1.6 mg) at t_R_ = 8.6 min) and compound **7**; monocerin (7.8 mg) at t_R_ = 9.1 min. The mycelial crude extract was chromatographed in a similar manner as described above, yielding 77.8 mg of deoxyphomalone (**8**, t_R_ = 11.2 min) but none of the other compounds.

### Evaluation of antimicrobial activities

Minimum inhibitory concentrations (MIC) of compounds **1**–**8** were determined in serial dilution assays against *Bacillus subtillis* DSM10, *Mucor plumbeus* MUCL 49355 and *Candida tenuis* MUCL 29892 as described previously by Chepkirui et al. (2016). The assays were carried out in 96-well microtiter plates in YMG (yeast-malt-glucose) medium for filamentous fungi and yeasts and MH (Müller-Hinton) medium for the bacterium. For all tested compounds, the starting concentration was 100 µg/mL and final 0.78 µg/mL.

### Water agar plate assay

The fungal cultures were tested in the water agar plate assay against *Caenorhabditis elegans* nematodes (wild type strain, see Ashrafi et al. 2017), in a similar manner as previously described by Stadler et al. (1994). After 3–7 days, nematicidal effects became visible by many dead and immotile nematodes in the vicinity of the mycelia. Fungal colonies exhibiting toxic effects were selected for submerged cultivation and production/isolation of nematicidal compounds.

### Microtiter plate assay for nematicidal activities

The nematicidal activity against *C. elegans* of all isolated compounds was determined by a slightly modified method (Stadler et al. 1994, Kuephadungphan et al. 2017 and Ashrafi et al. 2017). *C. elegans* was inoculated monoxenically on nematode agar (soy peptone 2 g/l, NaCl 1 g/l, agar-agar 20 g/l) and, after autoclaving, the following ingredients were added as sterile filtered solutions: cholesterol (1 mg/mL dissolved in EtOH) 0.5 ml, 1M CaCl_2_ 1 mll, 1M MgSO_4_ 1 ml, 40 mM potassium phosphate buffer 12.5 ml; pH 6.8) with living *Escherichia coli* DSM498 (1 ml of a suspension containing approximately 10 cells/ml, pre-inoculated for 12 h at 37 °C) and the plates were incubated at 21 °C for 4–5 days. Thereafter, nematodes were washed down from the plates with M9 buffer (3 g KH_2_PO_4_, 6 g Na_2_HPO_4_, 5 g NaCl and, after autoclaving, the addition of 1 ml 1 M MgSO_4_). Finally, a nematode suspension of approximately 500 nematodes/ml in M9 buffer was prepared and used in the microtiter plate assay.

The assay was performed in 24-well microtiter plates at four concentrations (100, 50, 25 and 12.5 µg/ml) for each compound. Ivermectin was used as a positive control, while methanol was used as a negative control. The plates were incubated at 20 °C in the dark and nematicidal activity was recorded after 18 h of incubation and expressed as LD_90_ (i.e. concentration causing over 90 % immobility of the nematodes).

### Antifungal activity assay against *Phellinus tremulae*

Growth inhibition of *Phellinus tremulae* CBS 123.40 for compounds 1–8 was tested according to the modified protocol published by Ayer and Jimenez (1994). The assay was performed in 24-well microtiter plates where 1 mL of YM agar was added in each well and thereafter the compounds were dissolved in methanol (100, 50, 25 and 12.5 µg/ml) and added to the wells. Shortly after the media solidified, 0.5 × 0.5 mm^2^ agar plugs of actively growing colonies of *Ph. tremulae* CBS 123.40, grown on a YM 6.3 agar plate, were placed in each well of the microtiter plate. Nystatin and methanol were used as positive and negative controls, respectively, together with control wells without additives. Inhibition of the radial growth of the colonies of *Ph. tremulae* CBS 123.40 relative to the control was recorded as a positive result. The radial growth was measured after 3, 5, 7 and 9 d.

### Phytotoxic activity assay

Phytotoxic activities were carried out by germination and seedling growth bioassay against *Setaria italica* and *Lepidum sativum* according to the protocol from Anke et al. (1989). The amount of 100 µg/paper disc of compound was tested; as a positive control herbicide methyl vilogen dichloride hydrate was used. The negative controls were the seeds only and the solvent alone (the one used for dissolving the compounds).

## Results and discussion

### Molecular phylogenetic analysis

**Figure 1.**
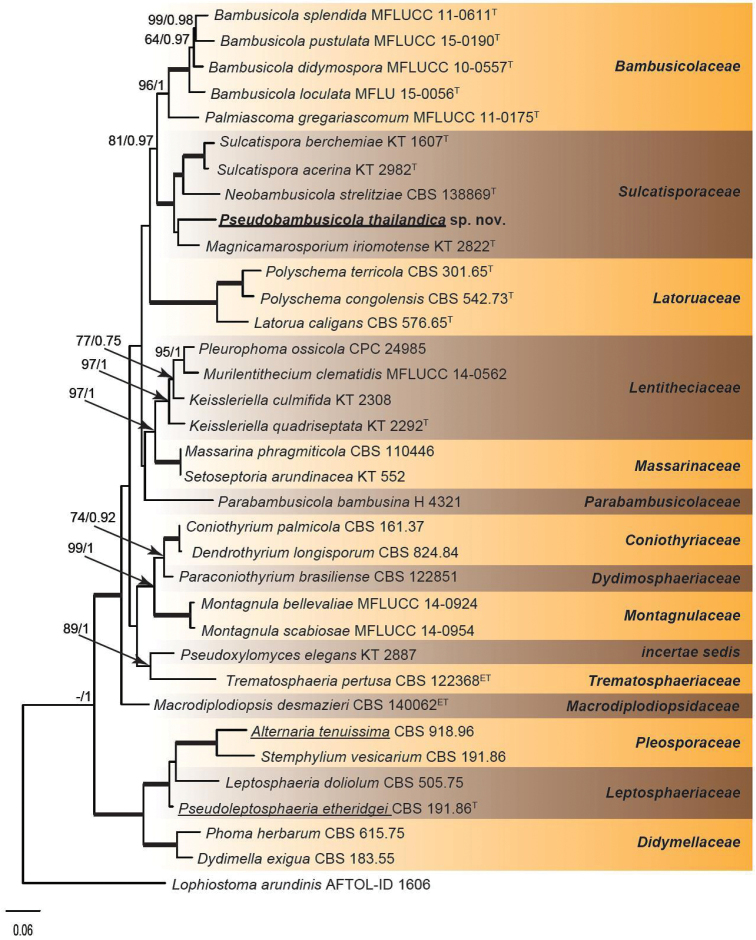
Phylogenetic tree (RAxML) inferred from the DNA sequence data of four loci (ITS, LSU, *tef1* and *rpb2*) of *Pseudobambusicola thailandica* and related species in Pleosporales (Dothideomycetes). The new taxon is indicated in **bold**. Taxa reported to produce deoxyphomalone are indicated by an underlined. Maximum likelihood bootstrap values ≥ 70 % and Bayesian posterior probabilities ≥ 0.95 are shown at the nodes and the scale bar indicates the number of expected mutations per site. Clades with 100 BML and 1 PP are indicated by thickened lines . The tree was rooted to *Lophiostoma arundinis* (AFTOL-ID 1606). ^T^ = ex-type strain; ^ET^ = epitype strain.

The combined dataset consisted of 35 taxa with 3126 characters of which 396 bp corresponded to ITS, 853 bp to LSU, 904 bp to *rpb2* and 973 bp to *tef1*. The alignment had 100% representation for LSU, 74% for ITS, 46% for *rpb2* and 57% for *tef1*. The phylogenetic tree (Fig. 1) shows two fully supported main clades, corresponding to the sub-orders Massarineae and Pleosporineae (Pleosporales, Dothideomycetes). In the Massarineae, eleven clades representing families are shown, i.e., Bambusicolaceae (96%, 1 pp), Coniotyriaceae (100%, 1 pp), Didymosphaeriaceae, Latoruaceae (100%, 1 pp), Lentitheciaceae (97%, 1 pp), Macrodiplodiopsidaceae, Massarinaceae (100%, 1 pp), Montagnulaceae (100%, 1 pp), Parabambusicolaceae, Sulcatisporaceae (100%, 1 pp) and Trematosphaeriaceae and an additional subclade comprising *Pseudoxylomyces elegans*. In the phylogenetic tree (Fig. 1), the sequence data of the new species indicate a systematic position in an independent branch in the Sulcatisporaceae close to *Magnicamarosporium eriomotense* without any support.

### Taxonomy

#### Pseudobambusicola

Taxon classificationFungiPleosporalesSulcatisporaceae

Hern.-Restr. & Crous gen. nov.

MB824299

##### Etymology.

The name reflects its morphological similarity of the type species to the asexual morphs of *Bambusicola* and *Neobambusicola*.

##### Type species.

*Pseudobambusicola thailandica* Hern.-Restr. & Crous.

##### Diagnosis.

Differs from *Neobambusicola* in having conidiomata with a neck, the presence of globose to subglobose thick-walled cells adjacent to the conidiomata and the production of chlamydospores in culture.

*Mycelium* composed of hyaline to pale brown, septate, smooth to slightly verruculose, hyphae. *Conidiomata* pycnidial, semi- or entirely immersed in the agar, solitary or aggregated, erumpent, globose with a neck, opening via central ostiole, dark brown, surrounded by dark brown, smooth to slightly verruculose hyphae, at the base globose to subglobose, thick-walled cells often present. *Conidiophores* reduced to conidiogenous cells. *Conidiogenous cells* phialidic with periclinal thickening at the conidiogenous locus, subcylindrical to ampulliform, hyaline, smooth. *Conidia* exposed in white, mucous drops at the ostioles of the pycnidia, composed by macro- and microconidia. *Macroconidia* produced in white, mucous heads, solitary, fusoid-ellipsoid, apex bluntly to subobtusely rounded, tapering to a distinctly truncate base, prominently guttulate, hyaline, smooth, 0–3-septate. *Microconidia* produced in the same pycnidia as macroconidia, solitary, oblong to cuneiform, non-guttulate to slightly guttulate, hyaline, smooth, aseptate. *Chlamydospores* brown, terminal at the tips of vegetative hyphae, in chains. *Sexual morph* not observed.

#### Pseudobambusicola thailandica

Taxon classificationFungiPleosporalesSulcatisporaceae

Hern.-Restr. & Crous sp. nov.

MB824300

##### Etymology.

The epithet refers to Thailand, where this species was collected.

##### Type.

THAILAND. Lop Buri Province: Chai Badan, Wang Kan Lueang Arboretum, Wang Kan Lueang Waterfall, on twig (unidentified), 14 Jul 2015, M. Hernández-Restrepo, MHR 1534 (holotype: BBH 42022!, culture ex-type BCC 79462!).

##### Description of fungal structures on SNA.

*Mycelium* composed by hyaline to pale brown, septate, smooth to slightly verruculose, hyphae, 1–2.5 µm wide. *Conidiomata* pycnidial, semi- or entirely immersed in the agar, solitary or aggregated, erumpent, globose, sometimes with a neck, opening via central ostiole, dark brown, 63–360 µm diam., sometimes with a cylindrical neck 50–125 × 40–50 µm, opening via central ostiole; at the base of the conidiomata are often present globose to subglobose cells, thick-walled, 5–9 µm wide; conidiomata surrounded by dark brown, smooth to slightly verruculose hyphae, 2–2.5 µm wide. *Conidiophores* reduced to conidiogenous cells. *Conidiogenous cells* phialidic with periclinal thickening, subcylindrical to ampulliform, hyaline, smooth, 6.5–7 × 2.5–4 µm. *Conidia* exposed in white, mucous drops at the ostiole of pycnidia, composed by macro- and microconidia. *Macroconidia* produced in white, mucous heads, solitary, fusoid-ellipsoid, apex bluntly to subobtusely rounded, tapering to a distinctly truncate base, mostly straight, but sometimes slightly curved, prominently guttulate, hyaline, smooth, 0–3-septate, 10–20 × 2–4(–6) µm. *Microconidia* produced in the same pycnidia with macroconidia, solitary, oblong to cuneiform, non-guttulate to slightly guttulate, hyaline, smooth, aseptate, 2–4(–5.5) × 1–2 µm, apex rounded, base truncate. *Chlamydospores* brown, terminal, in chains, 16–38 × 5–6 µm. *Sexual morph* not observed.

##### Culture characteristics.

Colonies on OA at 25 °C reaching 24 mm diam. in 2 weeks, elevated, with dense cottony mycelium at the centre, mouse grey, margin whitish, effuse to fimbriate; reverse dark mouse grey.

##### Notes.

*Pseudobambusicola* is introduced here for a pycnidial coelomycete producing two kinds of conidia. Morphologically, it is similar to the species of *Bambusicola* and *Neobambusicola*. However, asexual morphs in *Bambusicola* are characterised by brown or pale brown conidia and annellidic rather than phialidic conidiogenous cells and hyaline conidia as in *Pseudobambusicola* (Dai et al. 2012, 2017). *Neobambusicola* is a monotypic genus erected for *N. strelitziae*, first described from South Africa growing on necrotic leaf tissue associated with *Phyllachora strelitziae* (Phyllachoraceae, Phyllachorales, Sordariomycetes) (Crous et al. 2014). Both genera are similar in having pycnidial conidiomata and phialidic conidiogenous cells that produce fusoid-ellipsoid macro- and subcylindrical microconidia. However, in the new genus, the conidiomata are surrounded by dark brown, smooth to slightly verruculose hyphae and, in mature conidiomata, a cylindrical neck is often present; furthermore, chlamydospores can be present in culture. Although both genera belong to the Sulcatisporaceae (Pleosporales, Dothideomycetes), they are placed in different clades, *Neobambusicola* is more closely related to *Sulcatispora* (100 %, 1 pp), while *Pseudobambusicola* was placed in a distinct branch with *Magnicamarisporium* (Fig. 1). Additionally, based on LSU, ITS and *tef1* sequences, *P. thailandica* is 97 % (KP004495) and 83 % (KP004467) and 93 % similar to *N. strelitziae*, respectively.

**Figure 2.**
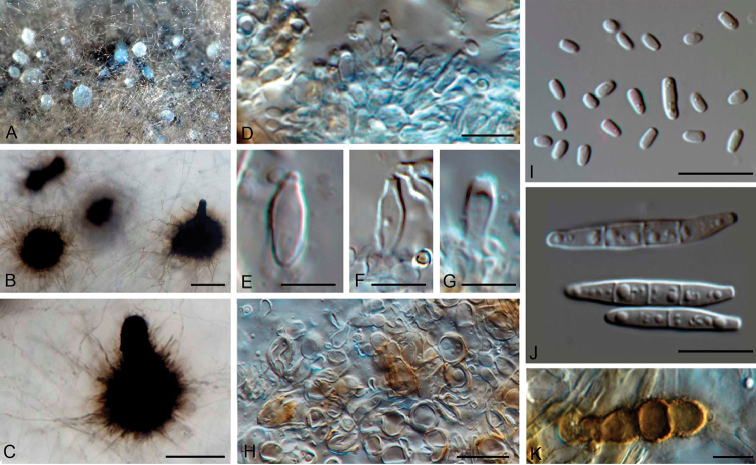
*Pseudobambusicola thailandica* (BCC 79462) on SNA. **A** Colony overview **B–C** Pycnidia **D–G** Conidiogenous cells **H** globose to subglobose cells, thick-walled, at the base of the conidiomata **I** Microconidia **J** Macroconidia **K** Chlamydospores. Scale bars: 200 µm (**B**), 100 µm (**C**), 10 µm (**D, H, I–K**), 5 µm (**E–G**).

### Water agar plate assay

Out of 66 fungal strains investigated, 18 exhibited antagonistic activity towards nematodes in the water agar assay. Of those, 3 strains produced compounds with nematicidal activity in submerged culture, while in 5 strains, antimicrobial activity was observed. Extracts from *P. thailandica* (BCC 79462) submerged fermentation displayed strong activity towards nematodes and were subjected to extensive chromatographic studies as described in the Experimental part.

### Structure elucidation of compounds 1–8

**Figure 3.**
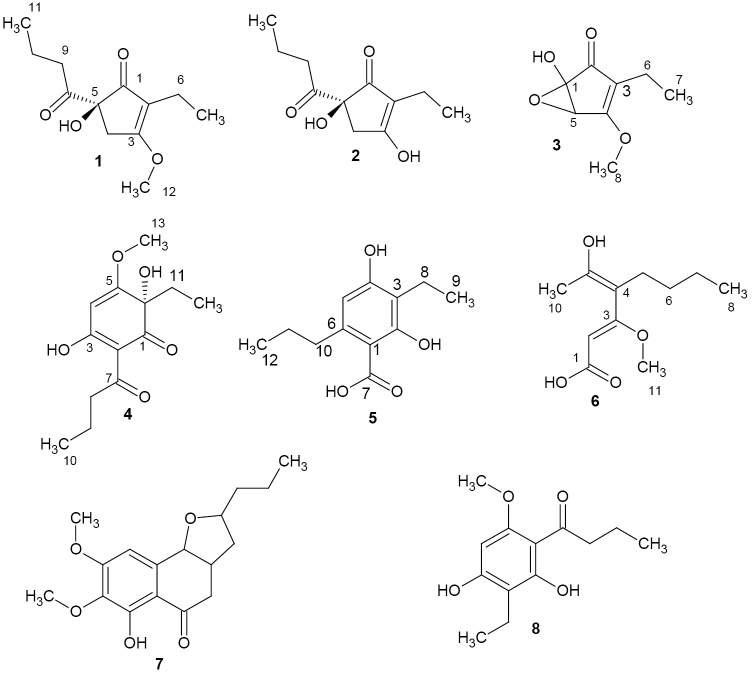
Chemical structures of thailanones A–F (**1**–**6**), monocerin (**7**) and deoxyphomalone (**8**).

Fractionation of the crude extracts obtained from submerged cultures of *P. thailandica* (BCC 79462) resulted in the identification of six previously undescribed polyketides for which the authors propose the trivial names thailanones A–F (**1**–**6**) and two known compounds, monocerin and deoxyphomalone, **7** and **8** (see chemical structures in Fig. 3). The NMR spectroscopic data are compiled in Tables 2 and 3 and the spectra and chromatograms are compiled in the Suppl. material 1.

Compound **1** (thailanone A) was isolated as a white solid from the supernatant with the molecular formula C_12_H_18_O_4_ and four degrees of unsaturation established from the HRMS data. ^13^C and DEPT NMR data revealed the presence of 12 carbons in the molecule: three methyl groups, four methylene groups and five quaternary carbons (Table 2). ^1^H NMR spectra on the other hand revealed the presence of two methyl triplets at δ 0.88 (H-11) and δ 0.93 (H-7) together with a methoxy group singlet resonating at δ 4.05 (H-12).

**Table 2.** NMR spectroscopic data for compounds **1**–**3** in D_6_-acetone (^1^H NMR at 700 MHz; ^13^C at 500 MHz).

	1			2			3	
No.	^13^C	DEPT	^1^H/HSQC	^13^C	^1^H/HSQC		DEPT	
1	202.6	C		202.6		83.9	C	
2	120.2	C		119.0		194.1	C	
3	187.9	C		191.2		120.3	C	
4	38.9	CH_2_	2.61 (s),3.29 (s)	43.8	2.41 (s)2.88 (s)	170.1	C	
5	86.3	C		84.4		58.3	CH	3.46 (s)
6	15.5	CH_2_	2.11 (q), J= 7.53 Hz	15.2	2.11 (q), J= 7.53 Hz	16.7	CH_2_	2.16 (q), J=7.53 Hz
7	12.7	CH_3_	0.93 (t), 7.53 Hz	12.8	0.93 (t), 7.53 Hz	13.2	CH_3_	0.78 (t), J=7.53 Hz
8	210.7	C		210.1		57.4	CH_3_	3.84 (s)
9	40.1	CH_2_	2.61 (m), 2.74(m)	38.9	2.53 (dt), J=7.10, 17.96 Hz2.68 (m), J=7.10, 17.96 Hz			
10	17.8	CH_2_	1.55 (m)	17.6	1.55 (m)			
11	14.0	CH_3_	0.88 (t), 7.42 Hz	13.9	0.86 (t), 7.31 Hz			
12	58.3	CH_3_	4.05 (s)					

**Figure 4.**
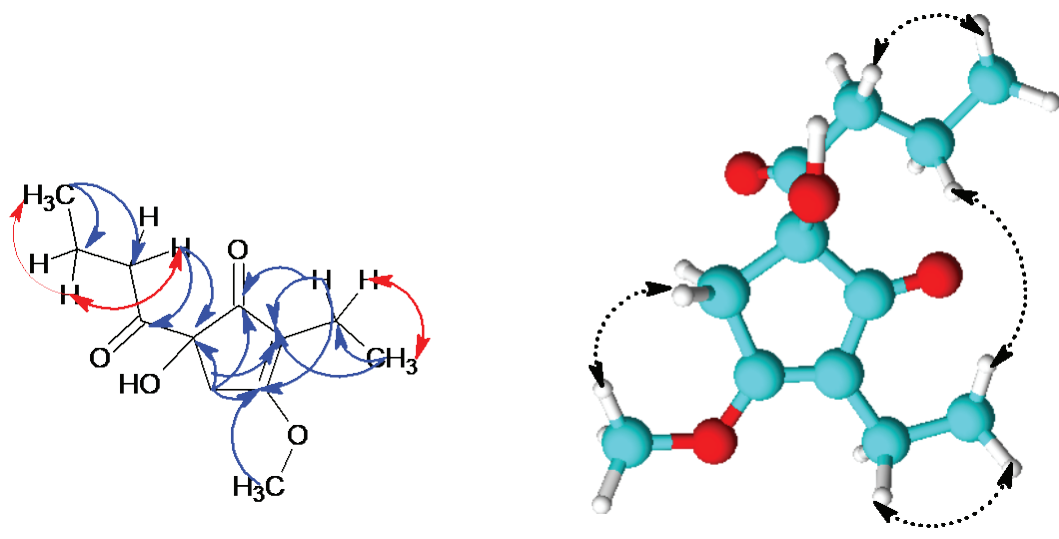
COSY, HMBC and ROESY correlations of **1.**

HMBC correlations of H-4 to C-1/C-2/C-3-C-5/C-6, H-6 to C-1/C-2/C-2/C-7 and H-9 to C-5/C-8/C-10/C-11 indicated the presence of an isohumulone moiety differing in the ring substitution (Fig. 4). Furthermore, HMBC correlations between H-11 to C-10/C-9 and H-7 to C-2/C-6 were observed. These correlations were further supported by the COSY correlations observed between H-10 and H-9/H-11 and H-6 and H-7. The methoxy proton H-12 showed a HMBC correlation to C-3. Therefore, the structure of compound **1** was established as 5-butanoyl-2-ethyl-5-hydroxy-3-methoxycyclopent-2-en-1-one.

Compound **2** (thailanone B) was obtained from the supernatant as a white solid. From the HR mass spectrum, its molecular formula was deduced as C_11_H_16_O_4_ with four degrees of unsaturation. Analysis of the ^1^H NMR and ^13^C NMR spectra of 2 suggested a closely related structure to that of 1 with the difference being the absence of the methoxy group at C-3. Further, the HMBC and COSY correlations observed were similar to those observed for 1. Hence, the structure was elucidated as (5S)-5-butanoyl-2-ethyl-3,5-dihidroxycyclopent-2-en-1-one.

The white solid compound **3** (thailanone C) with the molecular formula C_8_H_10_O_4_ and 4 degrees of unsaturation deduced from HR mass spectrum was isolated from the supernatant. The 1D and 2D NMR data of **3** suggested that the molecule has a closely related structure to **1** with one of the side chains missing. Analysis of the ^1^H NMR spectrum indicated the presence of a triplet at δ 0.78 (H-7) and a singlet at δ 3.84 (H-8) for methyl and methoxy groups, respectively. A COSY correlation was observed between H-6/H-7. Further, H-7 exhibited HMBC correlations to C-3/C-6, while H-6 was correlating to C-2/C3/C-4/C-7 in the HMBC spectra. H-5 on the other hand showed HMBC correlations to C-1/C-2/C-3/C-4. The epoxide ring was assigned based on the chemical shifts of C-1 (δ 86.3) and C-5 (δ 58.3) and also the established molecular formula. The methoxy group showed HMBC a correlation to C-4 (δ 170.1). The structure of **3** was established as 3-ethyl-1-hydroxy-4-methoxy-6-oxabicyclo[3.1.0]hex-3-en-2-one.

Compound **4** (thailanone D) was isolated as white solid. The molecular formula C_13_H_18_O_5_ was deduced from the HRMS data. ^13^C and DEPT NMR data indicated the presence of two methyl groups, a methoxy group, three methylene groups and five quaternary carbons in the molecule (Table 3). The ^1^H NMR spectra revealed 2 methyl triplets at δ 0.79 (C-12) and δ 0.96 (C-10). In addition, a methoxy singlet was observed at δ 3.94 (C-13). Networks of COSY correlations were observed between H-9 and H-8/H-10 and H-11 to H-12. In the HMBC spectra, H-4 showed correlations to C-2/C-3/C-5/C-6. Proton H-8 was correlating to C-2/C-7/C-9 /C-10, while the methyl protons H-10 were correlating to C-8/C-9 in the HMBC spectra. Further, H-11 showed HMBC correlations to C-1/C-5/C-6/C-12. The hydroxy group singlet at δ 4.44 showed HMBC correlations to C-1/C-5/C-6/C-11. Cross peaks in the ROESY spectra between the methoxy proton H-13 (δ 3.94) and the aromatic proton H-4 (δ 5.56) were observed. No ROESY correlations were observed between the hydroxy group proton (δ 4.44) and H-11/H-12/H-13. Hence the relative stereochemistry at C-6 can be assigned as S. The structure of compound **4** was elucidated as (6S)-2-butanoyl-6-ethyl-3,6-dihidroxy-5-methoxycyclohexa-2,4-dien-1-one.

**Table 3.** NMR spectroscopic data for compounds **4**–**6** in D_6_-acetone (^1^H NMR at 700 MHz; ^13^C at 500 MHz).

		4		5			6		
No.	^13^C	DEPT	^1^H/HSQC	^13^C	DEPT	^1^H/HSQC	^13^C	DEPT	^1^H/HSQC
1	196.36	C	–	104.2	C		164.3	C	
2	106.4	C	–	164.7	C		88.7	CH	5.42(s)
3	190.6	C	–	116.1	C		171.9	C	
4	96.0	CH	5.56 (s)	160.7	C		112.3	C	
5	177.9	C	–	111.1	CH	6.36(s)	25.0	CH_2_	2.35 (t), J=7.74 Hz
6	79.1	C	–	146.2	C		32.8	CH_2_	1.40 (p), J=7.31Hz
7	203.2	C	–	174.7	C		23.6	CH_2_	1.34 (m),
8	41.5	CH_2_	2.92 (m)	16.8	CH_2_	2.64 (q), J=7.53 Hz	14.7	CH_3_	0.91 (t), J=7.31 Hz
9	19.34	CH_2_	1.65 (m)	13.7	CH_3_	1.08 (t), J=7.53 Hz	159.3	C	
10	14.25	CH_3_	0.96 (t), J=7.31Hz	39.3	CH_2_	2.86 (t), 7.31Hz	17.7	CH_3_	2.19 (s)
11	36.12	CH_2_	1.79 (dq), J=7.53, 13.34 1.92 (dq), J=7.53, 13.34	26.0	CH_2_	1.59 (sext), J=7.31 Hz			
12	8.2	CH_3_	0.79 (t), J=7.53	14.6	CH_3_	0.93 (t), J=7.31			
13	57.7	CH_3_	3.94 (s)						
		OH	4.44 (s)						

The white solid compound **5** (thailanone E) showed the molecular formula C_12_H_16_O_4_ as deduced from HRMS data. The 1D and 2D NMR data of 5 suggested a closely related structure to 4 with the difference being in the ring substitution: The C-7 to C-10 chain and the carbon resonating δ 79.1 in 4 were missing. Analysis of the COSY spectra revealed correlations of H-8 to H-9 and H-11 to H-10/H-12. HMBC correlations of H-5 to C-1/C-3/C-4/C-10, H-8 to C-2/C-3/C-4/C-9, H-10 to C-1/C-5/C-6/C-11/C-12 and H-12 to C-10/C-11 were observed. Hence, the structure of the compound 5 was elucidated as 3-ethyl-2,4-dihydroxy-6-propylbenzoic acid.

Compound **6** (thailanone F) was obtained from the supernatant as a white solid with the molecular formula C_11_H_18_O_4_ established from HRMS data. Analysis of the ^1^H NMR data revealed a methyl group triplet at δ 0.91 (H-8), a methyl group singlet at δ 2.19 (H-10) and a methoxy singlet at 3.87 (H-11). HMBC correlations of H-2 to C-1/C-3/C-4 and H-10 to C-4/C-9 were recorded. Furthermore, HMBC correlations between H-5 and C-3/C-4/C-6/C-7/C-9, H-6 and C-4/C-5/C-7/C-8, H-7 and C-5/C-6/C-8 and H-8 and C-6/C-7 were observed. These correlations were further supported by the COSY correlations of H-6 to H-5/H-7 and H-7 to H-6/H-8. Cross peaks between H-2 (5.42) and methoxy protons H-11 (δ 3.87) were not observed in the ROESY spectra, indicating that the olefinic bond at position two had E configuration. The olefinic bond between C-4 (δ 112.3) and C-9 (δ 159.3) was assigned E configuration, since H-5 (δ 2.35) and H-10 (δ 2.19) also did not correlate in the ROESY spectra. The structure of the compound 6 was established as (2Z, 4E)-4-(1-hydroxyethylidene)-3-methoxyoct-2-enoic acid.

Monocerin (**7**) and deoxyphomalone (**8**) were identified by comparing their NMR and HRMS data with those reported in literature (Aldridge et al. 1970, Ayer and Jimenez 1994, respectively). Monocerin was reported before as a potent herbicide and insecticide against Johnson grass and woolly aphids, respectively (Grove et al. 1979, Robeson et al. 1982), while deoxyphomalone has been reported from other pleosporalean fungi like *Alternaria*. To the best of the authors’ knowledge, it has not been reported previously from a species of the Sulcatisporaceae.

### Biological activity

The results of the biological assays that were performed to detect antibacterial, antifungal and nematicidal activities are summarised in Table 4. Compound **6** was moderately active against *M. plumbeus* with MIC of 25 µg/ml, while deoxyphomalone (**8**) exhibited moderate activities against *B. subtilis* and *M. plumbeus* with MIC values of 12.5 and 25 µg/ml, respectively. Compounds **1**–**6** and **8** were also the only compounds with significant activities against *M. plumbeus.* The results by Ayer and Jimenez (1994), regarding the antifungal activity of phomalone and its deoxy derivative, were also repeated in the serial dilution assay. Compounds **2**–**7** failed to significantly inhibit growth of *Ph. tremulae*, whereas the new phomalone derivative **1** showed moderate inhibition and was more weakly active than the known compound **8**. No phytotoxic effects in plant germination and seedling growth bioassay with *S. italica* and *L. sativum* at 100 μg/paper disk were observed for any of tested compounds.

**Table 4.** Biological activities of compounds **1**–**8**.

Compounds	Antimicrobial activity MIC (µg/mL)		Nematicidal activity LD_90_ (µg/mL)	Antifungal activity (% growth inhibition at ≤ 12.5 µg/mL)
	*Bacillus subtillis* DSM10	*Mucor plumbeus* MUCL 49355	*Caenorhabditis elegans*	*Phellinus tremulae* CBS 123.40
Thailanone A (**1**)	≤ 50	–	≤ 50	50
Thailanone B (**2**)	–	–	≤ 25	28.6
Thailanone C (**3**)	–	–	≤ 25	31.4
Thailanone D (**4**)	–	≤ 25	≤ 12.5	28.6
Thailanone E (**5**)	–	–	≤ 50	25.
Thailanone F (**6**)	–	–	≤ 25	41.4
Monocerin (**7**)	–	–	–	28.6
Deoxyphomalone (**8**)	≤ 12.5	≤ 25	≤ 12.5	50
*Standards*				
Nystatin ^#^	–	≤ 0.782	–	100
Ciprofloxacin ^††^	≤ 0.78	–	–	–
Ivermectin ^‡‡^	–	–	≤ 12.5	–
Methanol	–	–	–	–

No activity against Candida tenuis, Setaria italica and Lepidum sativum was observed for any of tested compounds up to concentrations of 100 µg/mL. # Nystatin-antifungal reference; †† Ciprofloxacin-antibacterial reference; ‡‡ Ivermectin-nematicidal reference.

Compound **7** was reported as a potent herbicide and insecticide against Johnson grass and woolly aphids, respectively (Grove et al. 1979, Robeson et al. 1982). It was first isolated from a fungus described as *Phoma etheridgei* (Hutchison et al. 1994) and recently, **7** was also isolated from *Alternaria tenuissima* (Pleosporaceae) and the bioactivity was tested against *E. coli* (Anyanwu and Sorensen 2015). It has previously been reported to be active against the pathogenic basidiomycete *Phellinus tremulae*, which infects different species of poplar (Ayer and Jimenez 1994). This fungus causes extensive damage to hardwoods in North America and, in Canada, *Ph. tremulae* seriously reduces the economic value of *Populus tremuloides* (Trifonov et al. 1992). This prompted the authors to re-evaluate the compound in an antifungal assay against *Ph. tremulae*, in which all purified metabolites from *P. thailandica* were tested.

Moreover, the phytotoxic activity of terrein and congeners on plant growth and induction of lesions on fruit surfaces were previously investigated by Zaehle et al. (2014). Terrein, a major metabolite of *Aspergillus terreus*, resembles **1** and **7** in its chemical structure. The authors therefore performed a phytotoxicity assay, but they did not find significant effects of these compounds on germination and shoot/root elongation.

## Conclusion

In the course of this investigation of the fungal specimens collected in the rainforest of Thailand, several nematode-antagonistic strains were detected. The use of nematodes as test organisms can detect bioactivity from the compounds that are not detected by whole-cell-based screening for antimicrobial activities. As an outcome of the antihelmintic screening, six new compounds (thailanones 1–6) and two known compounds, deoxyphomalone (7) and monocerin (8) were isolated and further evaluated regarding their antifungal activity. Even though these results are just preliminary and the biological activities of the new compounds are rather moderate, they are very likely to play an important chemo-ecological role in the natural habitat of the fungal producer organisms, e.g. to protect against nematode predation. The authors have not yet tried to detect the metabolites on water agar in the presence of nematodes because of the experimental limitations that would first need to be overcome. The moderate activity of the new compounds (as compared to, for example, the standard ivermectin, which is at least ten times more active) probably precludes their adoption as a nematicidal agent that could serve as a candidate for an antihelmintic drug or an agrochemical nematicide. On the other hand, the fungus might turn out to be a candidate for a biocontrol agent to act as an antagonist of pathogenic nematodes and fungi.

## Supplementary Material

XML Treatment for Pseudobambusicola

XML Treatment for Pseudobambusicola thailandica
